# Impact of COVID-19 program adaptations on costs and cost-effectiveness of community management of acute malnutrition program in South Sudan

**DOI:** 10.1017/S1368980023002719

**Published:** 2023-12-14

**Authors:** Kemish Kenneth Alier, Hannah Tappis, Sule Ismail, Shannon Doocy

**Affiliations:** 1 Center for Humanitarian Health, Johns Hopkins Bloomberg School of Public Health, Baltimore, MD 21205, USA; 2 US Centers for Disease Control and Prevention, Juba, South Sudan; 3 Integral Global Consulting, Atlanta, GA

**Keywords:** Acute malnutrition, Community management of acute malnutrition, Treatment costs, Cost-effectiveness, COVID-19, South Sudan

## Abstract

**Objective:**

We assessed the impact of the COVID-19 pandemic and the protocol adaptations on cost and cost-effectiveness of community management of acute malnutrition (CMAM) program in South Sudan.

**Design:**

Retrospective program expenditure-based analysis of non-governmental organisation (NGO) CMAM programs for COVID-19 period (April 2020–December 2021) in respect to pre-COVID period (January 2019–March 2020).

**Setting:**

Study was conducted as part of a bigger evaluation study in South Sudan.

**Participants:**

International and national NGOs operating CMAM programs under the nutrition cluster participated in the study.

**Results:**

The average cost per child recovered from the programme declined by 20 % during COVID from $133 (range: $34–1174) pre-COVID to $107 (range: $20–333) during COVID. The cost per child recovered was negatively correlated with programme size (pre-COVID r-squared = 0·58; during COIVD r-squared = 0·50). Programmes with higher enrollment were cheaper compared with those with low enrolment. Salaries, ready to use food and community activities accounted for over two-thirds of the cost per recovery during both pre-COVID (69 %) and COVID (79 %) periods. While cost per child recovered decreased during COVID period, it did not negatively impact on the programme outcome. Enrolment increased by an average of 19·8 % and recovery rate by 4·6 % during COVID period.

**Conclusions:**

Costs reduced with no apparent negative implication on recovery rates after implementing the COVID CMAM protocol adaptations with a strong negative correlation between cost and programme size. This suggests that investing in capacity, screening and referral at existing CMAM sites to enable expansion of caseload maybe a preferable strategy to increasing the number of CMAM sites in South Sudan.

Acute malnutrition is a major global public health problem. Of the estimated 45·4 million children under 5 years wasted globally at any time in 2020, 27 % of which are in Africa^(1)^. The COVID-19 pandemic increased the burden of undernutrition, leading to 6·7 million (14·3 %) additional children being affected by wasting worldwide^(2)^. Meanwhile, the unprecedented global social and economic disruptions severely affected health systems^(3)^. Many countries struggled to ensure continuity of preventive and curative health services as they diverted resources to fighting the pandemic and health systems became overwhelmed, which limited access to critical services^(4)^. The COVID-19 pandemic could undo the progress made in fighting malnutrition due to the rising costs of goods and services, scarcity of supplies due to supply chain disruptions and a host of other factors that impact health service demand and care seeking^(5)^.

Protracted conflict and historical marginalisation have left South Sudan with weak infrastructure and governance and an under-resourced health system. Given the low national budget allocation to the health sector, over 80 % of healthcare services are funded through donor aid and contracted to international and national non-governmental organisations (NGO), that provide both technical and operational support, including paying for staff salaries^(6)^. Poverty as a driver of poor health outcomes is rampant, with two-thirds of South Sudan’s population living under $2·15/d^(7)^.

During the timeframe of the costing study and at the onset of COVID-19, South Sudan faced a food security crisis. An estimated 6·8 million people (55 % of the population) required food assistance, and six of the ten states were worse affected^(8)^. Despite South Sudan’s tremendous efforts in addressing acute malnutrition, evidenced by a reduction in the prevalence from 23 % to 12·6 % in the last decade, an estimated 1·3 million children between 6 and 59 months were acutely malnourished between January and December 2022^(8,9)^. Scaling up nutrition treatment sites, increasing by more than ten-fold between 2010 and 2022, has largely contributed to the reduction in the prevalence of malnutrition^(8)^. Like in many other countries, the COVID-19 pandemic has threatened to undo the progress in addressing undernutrition. The unintended consequences of COVID-19 prevention measures have increased child mortality from preventable causes due to vaccination and health services disruptions^(10)^.

Children with acute malnutrition are treated using the community management of acute malnutrition (CMAM) programs approach, which provides outpatient and in-patient care for most cases using specialised foods and regular health and nutrition monitoring. The South Sudan Nutrition Cluster modified the national CMAM guidelines, adapted in 2017, to ensure CMAM program continuity during the COVID-19 pandemic. The South Sudan CMAM protocol deviates slight from the WHO protocol, and it transfers SAM children to MAM protocol once their condition recovers from SAM to MAM.

The changes were in line with the Global Nutrition Cluster guidelines^(11)^, a global coordination mechanism led by UNICEF that is responsible for the overall nutrition programme coordination. The modifications are listed in Table 1 ^(12)^. In 2018, the South Sudan Nutrition Cluster conducted a CMAM cost analysis that assessed NGO CMAM program costs and aggregated nutrition program cost distribution at the state level and described bundled high-level cost drivers^(13)^. The 2018 cost analysis estimated treatment costs for children with severe and moderate malnutrition (SAM and MAM) at US$358 and US$63 per child, respectively, and observed the main cost drivers were technical support, programme management, nutrition supplies and logistics. With changes to nutrition treatment protocols during the pandemic, there was a need to understand the impacts of protocol modifications on nutrition programme costs and drivers. We anticipated that due to the changes in programme and the global supply chain challenges, the overall cost of providing CMAM could have risen during the pandemic. In a report of assessment conducted between 2020 and 2021, NGOs reported that hiring additional cars to ensure fewer staff used a car at a time, training of health workers on the adapted guidelines, supply chain and logistics could have increased programme costs, while virtual trainings which eliminate the need to rent training venues, use of caregivers to administer MUAC screening could reduce costs^(14)^. This paper examines if and how nutrition programme costs changed following the introduction of COVID-19 adaptations with the aim of informing programme implementation strategies and nutrition policy as the pandemic subsides.

**Table 1:** South Sudan CMAM guidelines adaptations

Category	Pre-COVID CMAM Guideline	Adaptation during COVID-19
Admission criteria	Admission criteria for SAM Bilateral pitting pedal oedema OR mid-upper arm circumference (MUAC) < 11·5 cm AND/OR weight for height/ length (WHZ/L) > –3 z-score. Admission criteria for MAM MUAC ≥ 11·5 < 12·5 cm OR weight-for-height/ length ≥ –3 < –2 z-score.	Suspend use of weight and height measurements and use simplified admission criteria (MUAC and/or oedema only) for both SAM and MAM.
Frequency of ration distributed	Weekly ration for SAM children and 2 weekly for MAM children.	Two weeks ration for SAM children and 4 weeks ration for MAM children.
Work schedule for outpatient therapeutic program (OTP) and targeted supplementary feeding program (TSFP)	OTP to be provided daily for new admissions and weekly or biweekly for revisits. TSFP to be provided daily for new admissions and revisits can be done on a specific day of the week/month.	Increase the number of days for OTP/ TSFP depending on number of beneficiaries and site capacity.
Appointments	Appointment based on individual ration schedule and days of when OTP/TSFP is open.	Adjust appointments for beneficiaries to come on different days of the week.
Outreach	Large community outreach or mobile programs.	Plan for mobile services closer to the communities for large villages (smaller and one on one sessions).
Active case finding (MUAC measurement)	Measuring of MUAC is conducted by trained health workers or community nutrition volunteers (CNV).	Mothers trained, issued with MUAC tapes and monitored to take MUAC measurements and self-refer in liaison with the CNV.
Discharge criteria	Discharge criteria from OTP MUAC discharge: MUAC ≥ 11·5 cm for at least two consecutive visits OR WHZ/L discharge: WHZ/L ≥ –3 z-score for at least two consecutive visits AND no bilateral pitting oedema for two consecutive visits AND child is clinically well and alert. Discharge criteria from TSFP MUAC discharge: MUAC ≥ 12·5 cm for 2 consecutive visits OR WHZ/L discharge: WHZ/L ≥ –2 z-score for 2 consecutive visits.	Discharge criteria from OTP MUAC ≥ 11·5 cm and < 12·5 cm child discharged and referred to TSFP. MUAC ≥ 12·5 cm child discharged with a two-week ration and a plan for weekly follow up visits by a CNV. Discharge criteria from TSFP MUAC ≥ 12·5 cm child discharged with a 2-week ration and a plan for weekly follow-up visits by a CNV.

## Methods

**Fig. 1:**
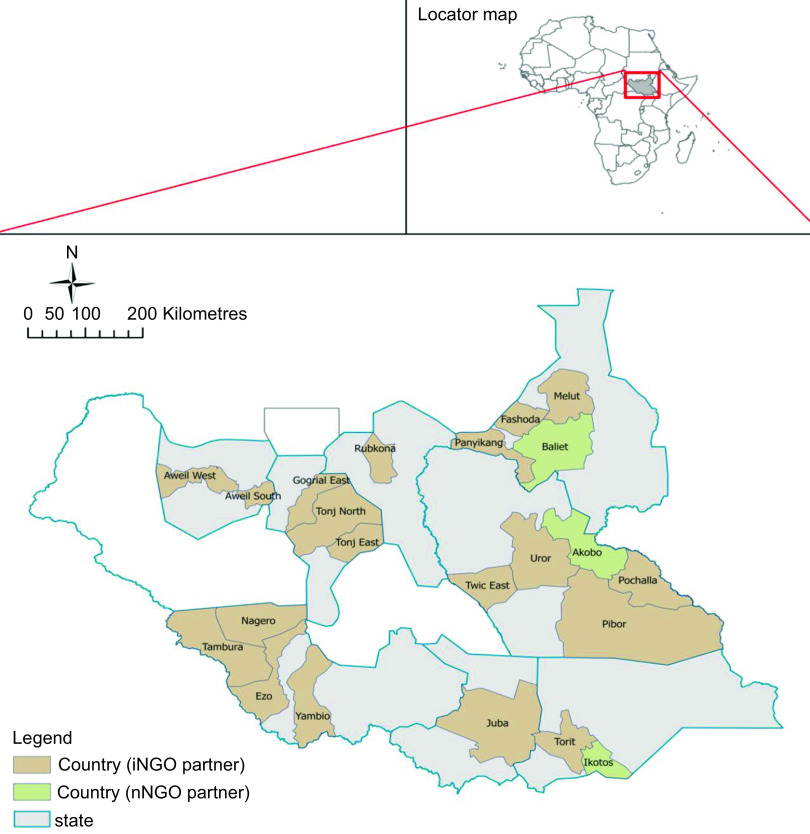
Map of included counties in the cost analysis (*n* 22)

This cost-effectiveness study was nested within a broader evaluation of COVID--19 nutrition program adaptations that explored the impact of the COVID-19 pandemic on CMAM programming^(15)^. We only considered NGO-associated cost for running CMAM programs based on original CMAM guidelines before COVID-19 and modified guidelines during COVID-19. Both guidelines were adapted based on World Health Organization and Global Nutrition guidelines^(16)^. The retrospective analysis compared nutrition program costs for two time periods – January 2019 to March 2020 (pre-COVID period) and April 2020 to December 2021 (COVID period). We conducted the study at the national level. After we presented the study at a nutrition cluster meeting, all NGOs operating in the country were invited to participate^(17)^. Nutrition program planning and implementation occur at the county level and the number of NGOs working in one county ranges between one and four depending on the county size, access constraints, funding and donor conditions. A total of eleven NGO nutrition partners volunteered to participate; collectively, these organisations operated in twenty two (28 %) of seventy-nine counties and eight of ten states (Fig. 1). We conducted data collection and analysis between November 2021 and June 2022.

The Food and Nutrition Technical Assistance (FANTA) project’s CMAM Costing Tool, Version 1.1 (2012), an excel-based tool for costing of community-based management of acute malnutrition^(18)^ was adapted to collect NGO cost expenditures for each of the two time periods. A KoboToolbox (KoBo, Inc.) questionnaire was used for electronic data collection of county-level costs for twelve expenditure categories: (1) medicines and medical/preventive supplies, (2) therapeutic food and supplements, (3) training and supervision, (4) logistics and transportation, (5) salaries, incentives and related cost, (6) fixed supplies, (7) infection prevention and control, (8) data management and communication, (9) community-level activities, (10) national level program management, (11) sub-national level program management and (12) evidence generation and assessments (see online supplementary material, Supplemental Table 1). For ease of analysis and comparison, we disaggregated and collapsed the following categories in Table 2: training and supervision category to training cost and supervision cost separately; logistics and transportation to transportation of supplies, storage of supplies and transportation of personnel; human resources was split into technical staff and non-technical staff and considered as a fixed cost. We collapsed national and sub-national management cost to programme management. The tool was presented to the nutrition partners, further refined for South Sudan context, and piloted by two NGO prior to use.

**Table 2:** Cost drivers per child recovered by program period

Category	Pre-COVID		COVID	
	Amount ($)	%tage	Amount ($)	%tage
Salaries (technical staff)*	43·0	32·4	33·3	31·2
Salaries (non-technical staff)*	10·9	8·2	6·5	6·0
Therapeutic food†	24·8	18·7	34·0	31·8
Community nutrition activities†	12·5	9·4	10·2	9·6
Fixed supplies*	8·2	6·2	2·3	2·2
Program management†	12·8	9·7	8·2	7·7
Transportation of supplies†	3·7	2·8	2·3	2·1
Storage of supplies†	1·8	1·3	1·5	1·4
Transportation of personnel†	1·5	1·1	1·0	0·9
Medicine and medical supplies†	4·9	3·7	3·1	2·9
Infection prevention and control†	3·5	2·7	1·4	1·3
Training†	1·4	1·0	0·7	0·6
Supervision†	1·4	1·0	1·0	0·9
Data management and communication†	1·6	1·2	1·0	0·9
Assessments and evidence generation†	0·5	0·4	0·4	0·4
Total	133		107	

* Fixed cost.

† Variable cost.

In coordination with the Nutrition Cluster, NGO partners were invited to four orientation sessions on the costing tool and programme and finance focal persons from NGO that expressed interest in participating were also identified. We emailed the electronic survey tool to the respective individuals with a request to submit one response for each county where the organisation was working. We used a WhatsApp group to provide real-time support to participating NGO staff during the data collection to ensure consistency, and one-on-one partner meetings were conducted with each participating organisation to provide further guidance and clarifications on the methodological approach.

Additionally, CMAM program enrollment and outcome data from the nutrition cluster were collected through the Nutrition Information System^(19)^. Data for SAM and MAM for children aged 6–59 months were used for computing cost efficiency and effectiveness. Total enrollment (new enrollment, moved-in and referrals) was used to compute cost efficiency, and the number of children discharged as recovered was used to calculate cost-effectiveness. For calculation of the recovery rate, the number of recovered children was used as the numerator and the total number of exits (recovered, defaulters, deaths, non-responders and medical transfer-out) as the denominator.

We used an ingredients-based approach to analyse CMAM program running costs incurred by NGOs during the specified periods. We considered cost for the overall CMAM program and did not differentiate by SAM or MAM because partners did not disaggregate budgets and expenditures by these categories. NGO programs were assumed to be similar in structure and quality, in line with the national guidelines. For medicines and ready-to-use therapeutic and supplementary foods (RUTF and RUSF, collectively referred to as RUF) that are provided as an in-kind donation to partners, we requested stock data from NGOs and used unit costs from the UNICEF supply division database to compute costs^(20)^. The following costs were not considered in the analysis: (1) amortisation value of investments prior to the data collection period, (2) major construction of service provision facilities and (3) logistical cost borne by the Logistics Cluster. Additionally, costs of malnutrition treatment incurred by households or direct government expenditures by the Ministry of Health were not considered.

Data analysis was conducted using Microsoft Excel software, version 16.63.1 (Microsoft Corporation, USA). Total programme costs were standardised to monthly costs to enable comparison of costs during the 15-month pre-COVID and 21 months ‘during COVID’ periods. Analyses examined total programme cost, programme cost per month, cost per child enrolled, cost per child recovered, cost drivers and recovery rates at the county level. All cost were calculated in United States Dollars (2021 USD). Cost per child enrolled and cost per child recovered were derived by dividing the total monthly cost of programme by the average monthly enrollment and recovered from the program. Aggregate state level analyses were computed by averaging findings across counties. Categorical data were summarised as frequencies and percentages, while numerical data were displayed as averages and ranges, differences between the pre-COVID and during COVID were assessed using percentage point differences.

This study was approved by the Johns Hopkins Bloomberg School of Public Health Institutional Review Board and South Sudan Ministry of Health Research Ethics Review Board.

## Results

**Fig. 2:**
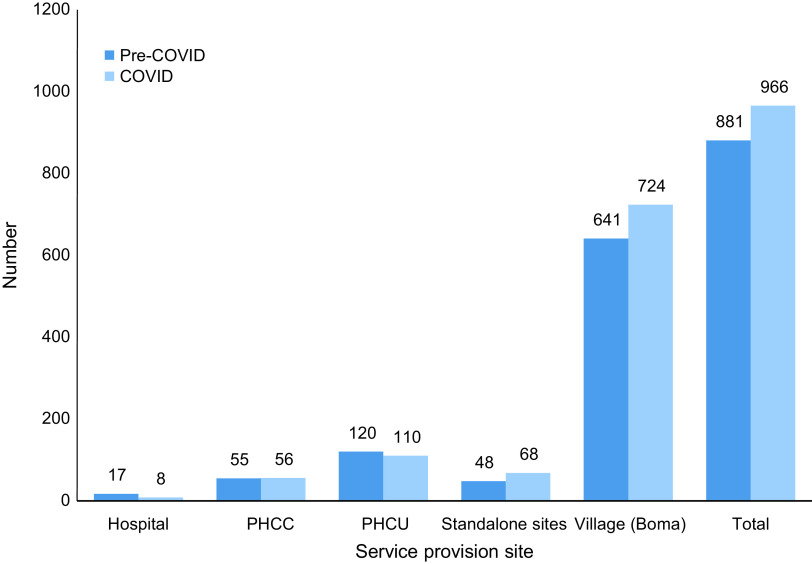
Nutrition Program Services by Site and Period (data for 11 NGOs in twenty-two counties). PHCC, primary health care centre (the frontline health facilities providing basic preventive and curative services with a catchment population of 15 000 people). PHCU, primary health care unit = (the first referral health facilities that offer a wider range of diagnostic and curative services with a catchment population of 50 000 people). Village (Boma): Community units where community health workers operate

CMAM program expenditures from eleven NGO partners, including nine international NGOs (iNGOs) and two national NGOs (nNGOs) operating in twenty-two counties spread across eight of the ten states in South Sudan (Fig. 1). The combined number of service provision sites (hospitals, primary health care centers, primary health care units and standalone sites) across all included NGOs remained relatively stable at 240 facilities pre-COVID and 242 facilities during COVID. However, there was a notable shift away from hospital-based service provision to lower-level sites and communities. The number of hospitals providing nutrition services decreased by 52 % during COVID (from seventeen pre-COVID to eight during COVID); meanwhile, the number of standalone and community sites providing nutrition services increased by 42 % and 13 %, respectively (Fig. 2).

### CMAM program enrollment and outcomes

The number of children enrolled in CMAM treatment programs increased by 19·8 % during COVID (Table 3) among organisations participating in the costing study, and there was a larger increase in enrollment among iNGO (20·7 %) as compared with nNGO (12·9 %). When comparing average monthly CMAM enrollment for the two time periods, a total of 12 240 children were enrolled in CMAM programs pre-COVID compared with 14 569 during COVID. When examined by state, only Central Equatoria (inclusive of Juba County) had a decrease in enrollment, which is not unexpected given that it is the capital city where COVID-19 restrictions were likely to more consistently enforced. The overall SAM:MAM treatment ratio was relatively constant over time at 0·5 SAM cases per MAM case both pre-COVD and during COVID. The highest SAM:MAM treatment ratio for both periods was in Jonglei state and the lowest in Unity. However, these figures should be interpreted with caution as not all counties in each state were included in the analysis.

**Table 3:** Program enrollment, SAM:MAM treatment ratio and recovery rates by period and organisation type

Number of counties	Overall	By state								By organisation type	
		Central Equatoria	Eastern Equatoria	Western Equatoria	Jonglei	N. Bahr el-Ghazal	Unity	Upper Nile	Warrap	iNGO	nNGO
	22	1	2	4	5	2	1	4	3	16	6
Total programme enrollment*											
Pre-COVID	176 361	14 790	12 104	8193	50 677	31 342	21 290	5920	32 045	155 142	21 219
During COVID	211 194	9000	14 181	21 290	59 548	31 666	30 010	9575	35 924	187 246	23 948
Change	19·8 %	−39·1 %	17·2 %	159·9 %	17·5 %	1·0 %	41·0 %	61·7 %	12·1 %	20·7 %	12·9 %
SAM:MAM treatment ratio											
Pre-COVID	0·5	0·4	0·5	0·4	0·7	0·5	0·3	0·4	0·7	0·5	0·5
During COVID	0·5	0·4	0·5	0·5	0·9	0·4	0·2	0·8	0·5	0·4	0·6
Change	0·0	0·0	0·0	0·1	0·2	0·0	−0·1	0·4	−0·2	−0·1	0·1
Recovery rate (average)											
Pre-COVID	90·2 %	86·5 %	87·7 %	88·9 %	87·1 %	89·9 %	94·8 %	88·0 %	90·8 %	92·4 %	84·9 %
During COVID	94·8 %	84·9 %	94·4 %	97·8 %	94·7 %	94·1 %	93·4 %	96·6 %	92·5 %	95·3 %	94·6 %
Change	4·6 %	−1·6 %	6·7 %	1·6 %	7·6 %	4·2 %	−0·3 %	8·6 %	1·7 %	2·9 %	9·7 %

* The data we analysed for total enrollment were standardised for each partner to equal number of months between the two periods (pre-COVID and COVID) to allow comparison.

Recovery rates improved during COVID by an average of 4·6 % and also became less variable in the twenty-two included counties. The average recovery rate (recovery rate for an NGO across all facilities they operate in the county) during pre-COVID was 90·2 % (range 69·2–98·2 %) compared with 94·8 % (range: 84·9–99·8 %) during COVID. When comparing iNGO and nNGO, average recovery rates were higher among iNGO both pre-COVID (92·4 % iNGO and 84·9 % nNGO) and during COVID (95·3 iNGO and 94·6 % nNGO). However, nNGO had greater gains in terms of improvement in the average recovery rate between the pre-COVID and COVID periods (2·9 % iNGO and 9·7 % nNGO), and during COVID recovery rates were relatively similar. Recovery rate analysed by state is limited because data were available for only one or two counties in four states. Additionally, it should be noted that recovery rates of organisations participating in the costing analysis often differed from county level recovery rates (see online supplementary material, Supplemental Table 3).

### Total programme costs

Total monthly CMAM program costs declined by 5·7 % during COVID period from $1 217 615 to $1 148 017 overall (Table 4). However, change in programme costs varied among states with cost increases in three states and cost decreases in the remaining five states. Costs decreased during the COVID period in Unity, Jonglei and Warrap by 18·6 %, 17·4 % and 10·7 %, respectively. The highest reductions were observed in Central Equatoria State (57·1 %), which includes the capital Juba, Eastern Equatoria (21·3 %) and Western Equatoria (16·2 %). nNGO had a notably higher reduction in programme costs (–15·8 %, range –24·2–17·3 %) compared with iNGO (–3·7 %, range –57·1–153·1 %).

**Table 4:** Program outcomes, recovery and costs by period and organisation type

	All counties	By state								By organisation type	
		Central Equatoria	Eastern Equatoria	Western Equatoria	Jonglei	N. Bahr el Ghazal	Unity	Upper Nile	Warrap	iNGO	nNGO
# of counties	22	1	2	4	5	2	1	4	3	16	6
Average monthly enrollment											
Pre-COVID	12 240	986	807	628	3442	2089	1774	395	2136	9404	2853
During COVID	14 569	600	945	115	2212	2111	2501	638	2395	10 336	4441
Change *n*	2329	−386	138	−513	−1230	22	727	243	259	932	1588
%	19 %	−39 %	17 %	−82 %	−36 %	1 %	41 %	62 %	12 %	10 %	56 %
Average monthly exits											
Pre-COVID	10 179	10 179	901	386	1662	1891	1644	260	1824	8079	2101
During COVID	11 374	11 374	538	632	2175	1824	1772	407	220	8529	2845
Change *n*	1195	1195	−363	246	513	−67	128	147	−1604	450	744
%	12 %	12 %	−40 %	64 %	31 %	−4 %	8 %	57 %	−88 %	6 %	35 %
Average monthly recovered											
Pre-COVID	9185	779	1079	373	1476	1680	1612	210	1651	7332	1852
During COVID	10 140	457	1789	614	2064	1698	1732	391	2024	8000	2704
Change *n*	955	−322	710	241	588	18	120	181	373	668	852
%	10 %	−41 %	66 %	65 %	40 %	1 %	7 %	86 %	23 %	9 %	46 %
Average monthly program costs											
Pre-COVID	$1 217 615	$176 963	$95 296	$182 463	$397 246	$132 776	$56 206	$71 665	$105 001	$1 010 876	$206 740
During COVID	$1 148 017	$75 878	$75 023	$152 846	$466 499	$124 190	$66 686	$70 689	$116 206	$973 913	$174 104
Change *n*	–$69 598	–$101 085	–$20 273	–$29 617	$69 253	–$8586	$10 480	–$976	$11 205	–$36 962	–$32 636
%	−6 %	−57 %	−21 %	−16 %	17 %	−6 %	19 %	−1 %	11 %	−4 %	−16 %
Cost per child enrolled											
Pre-COVID cost	$99	$179	$118	$291	$115	$64	$32	$182	$49	$94	$140
Range	27–609	*	88–136	170–431	62–150	52–85	*	93–609	27–64	88–166	27–609
During COVID cost	$78	$126	$79	$99	$115	$59	$27	$111	$49	$74	$104
Range	21–293	*	67–107	42–198	62–293	50–78	*	61–215	21–88	102–110	21–293
Change *n*	–$21	–$53	–$39	–$192	$0	–$5	–$5	–$71	–$1	–$19	–$35
%	−21 %	−30 %	−33 %	−66 %	0 %	−7 %	−16 %	−39 %	−1 %	−21 %	−25 %
Cost per child recovered											
Pre-COVID cost	$133	$227	$183	$489	$168	$79	$35	$341	$64	$123	$215
Range	34–1174	*	130–217	349–704	89–227	70–92	*	160–1174	34–85	130–358	34–1174
During COVID cost	$107	$166	$106	$249	$152	$73	$39	$181	$56	$102	$147
Range	20–333	*	99–117	186–323	73–330	66–86	*	91–333	20–121	117–200	20–333
Change *n*	–$26	–$61	–$77	–$240	–$17	–$6	$4	–$160	–$8	–$21	–$68
%	−20 %	−27 %	−42 %	−49 %	−10 %	−7 %	10 %	−47 %	−12 %	−17 %	−32 %

* State had only a single data point.

### Cost per child enrolled and recovered

Across all twenty-two counties, the average cost per child enrolled in a CMAM program was 21 % lower during the COVID period ($78, range: $21–293) compared with pre-COVID period ($99, range: $27–609). In the pre-COVID period, cost per child enrolled ranged from $291 to $32; in COVID period, costs per child enrolled ranged from $27 to $115. Compared with the pre-COVID period, all states experienced a reduction in cost per child enrolled, with marked decreases seen in four states ranging from 30 % to 66 %. nNGO had a higher reduction in cost of enrollment (25 %) compared with iNGO (21 %). While there was an overall reduction in cost per programme enrollment at the state level, some counties experienced increase in cost of programme per child enrolled (Table 4).

**Fig. 3:**
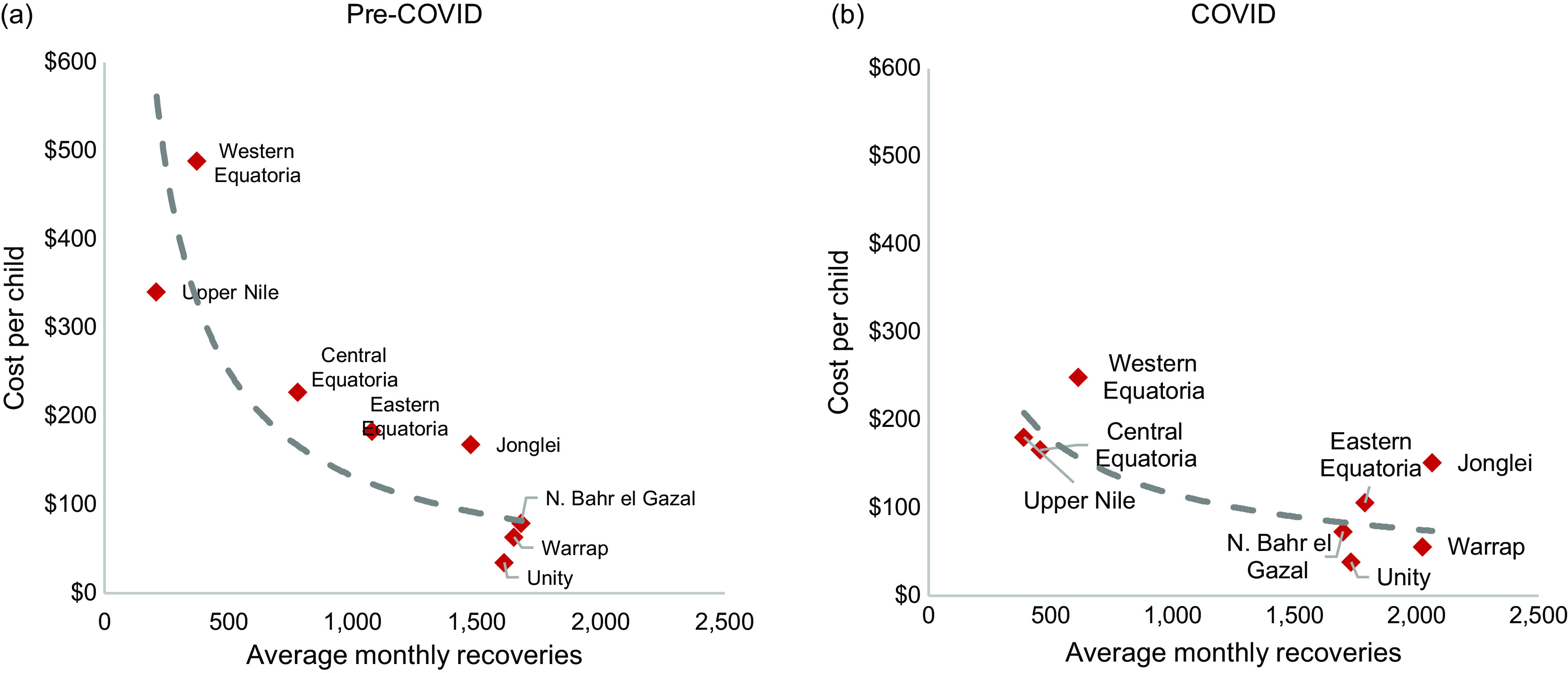
Cost per child recovered compared with programme size

Similarly, the average cost per child recovered declined by 20 % during COVID in the twenty-two counties analysed, from $133 (range: $34–1174) pre-COVID to $107 (range: $20–333) during COVID. The cost per child recovered ranged $35–$489 during pre-COVID and $59–$249 during COVID. Except for one state that had a 10 % increase in cost per child recovered, all the other states saw reductions in cost per child recovered during COVID, with the largest reduction of 49 %. There was a wide variability in cost per child recovered among counties within the same state and within the same partner that provided data for multiple counties. nNGOs had a larger decrease (32 %) in cost per child recovered, compared with iNGOs (17 % reduction). Cost per child recovered was negatively correlated with programme size (pre-COVID r-squared = 0·58; during COIVD r-squared = 0·50) (Fig. 3). In pre-COVID period, the state with the lowest number of children recovered from the program per month (373) was the most expensive programme per child recovered ($ 489), while the state with average monthly recovery of 1612 children was the cheapest programme ($35). During COVID, the trend was similar.

### Cost allocations (components)

**Fig. 4:**
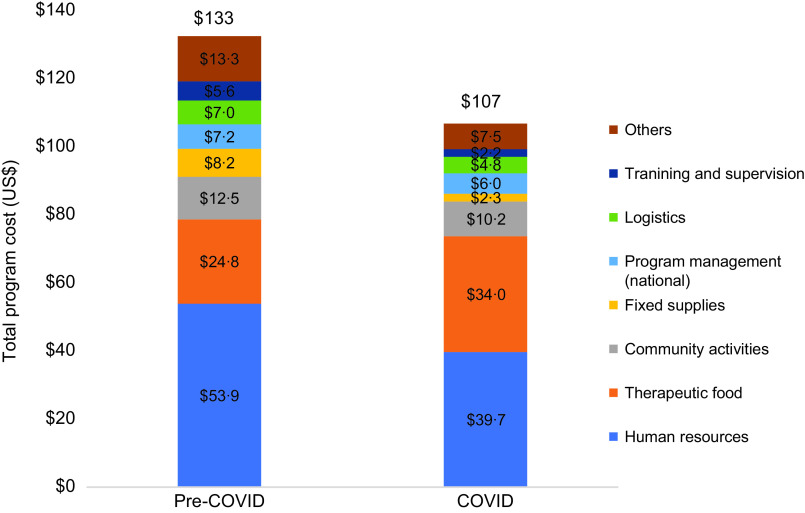
Nutrition Program Costs per Child Recovered, by category (pre-COVID and during COVID). Others, sub-national program management, medicine and supplies, infection prevention and supplies, data and communication and evidence generation

Salaries, RUF and community activities accounted for over two-thirds of the cost per child recovered during both pre-COVID (69 %) and COVID (79 %) periods (Fig. 4). Salaries alone accounted for more than half of costs pre-COVID and more than third during COVID. The contribution of the cost categories varied greatly by state. In the pre-COVID period, the contribution of salaries ranged from 14 to 65 %, RUF costs ranged from 0·1 to 32 % (there were massively low quantities of RUF reported by the NGOs during this period) and community activities ranged from 3 to 21 %. During COVID period, the contribution of salaries ranged from 21–64 %, RUF ranged from 4 to 47 % and community activities ranged from 3 to 23 %. Except for RUF where costs increased by 37 %, the absolute cost per child recovered decreased in all other cost categories examined. The greatest declines in cost per child recovered were in the provision of fixed supplies (72 %), medicines and medical supplies (61 %) and training and supervision (by 60 %).

## Discussion

In this retrospective cost-effectiveness study, we examined treatment costs for malnourished children in CMAM programs during the COVID-19 pandemic and compared with the pre-pandemic period. Acute malnutrition treatment costs decreased during the COVID-19 period with no negative impact on programme recovery rates. Additionally, the unit cost of treatment was negatively correlated with programme size. However, it is important to note that human resources cost was considered as a fixed cost, and we expect a stronger effect on unit cost.

Acute malnutrition treatment costs pre-COVID from this study, at $99 per child enrolled and $113 per child recovered, are comparable to other contexts. Acute malnutrition treatment costs have been estimated in Mali ($165)^(21)^, Ethiopia ($285 in a facility-based care setting and $135 in a community-based setting)^(22)^, Zambia ($203)^(23)^, Malawi (Base case $169·3, Best case $140·3 and worse case $211·6)^(24)^ and Bangladesh ($165)^(25)^. The Malawi analysis considered incremental costs of CMAM, while the Bangladesh study was conducted for a program implemented by only community health workers whose remuneration is lower than those of higher medical cadres. It should be noted that South Sudan uses a slightly modified WHO CMAM protocol where SAM children are shifted to MAM protocol after recovering from SAM to MAM. This deviates from the WHO protocol that provides for full treatment of SAM children under SAM protocol until full recovery. This full WHO protocol is for example used in Mali, and it may explain the higher unit cost in Mali compared with what we found in South Sudan given higher cost for RUTF used for SAM than RUSF used for MAM. Another likely driver of differences in costs is scale, where programme costs may be lower when caseloads are larger as was observed in our study. A cluster randomised trial conducted in South Sudan and Kenya^(26)^ found the cost of treatment per child recovered at $1041 and cost per child treated at $451. However, these were cross-country estimates, whereas the current study took place from South Sudan. We also included community activities of CMAM where community nutrition volunteers play a part in the continuum of care of SAM and MAM through referral, defaulter tracing and health education that improves seeking behaviours and increases treatment compliance. Additionally, a review conducted by Save the Children found that the cost per child treated for severe wasting ranged from $56 to $805, while cost per child recovered ranged from $114 to $1041^(27)^.

The predominant drivers of costs in this study were RUF, salaries and community activities. These findings are consistent with those in a CMAM program cost analysis conducted in South Sudan in 2018^(13)^ and other studies conducted in similar contexts. In Mali, personnel costs accounted for up to 56 % of treatment cost^(21)^ and in Bangladesh RUF comprised 30–40 % of treatment cost for similar programmes^(25)^. In our analysis, costs drivers varied widely across states (see online supplementary material, Supplemental Table 2) and were affected by state specific factors and shocks. Compared with pre-COVID period, RUF costs increased overall. NGOs reported that providing RUF for longer durations of time introduces risks of misuse and damage at the household level due to poor storage conditions^(28)^, leading to longer stays and using higher quantity of RUF to treat one child. This poor adherence when treatment follow-up duration is increased was reported in a study in Nigeria^(29)^. Additionally, due to the increase in food insecurity during the COVID period, anecdotal reports from NGOs indicated families did not have enough food, and RUTF and RUSF were shared with other siblings who were not enrolled in nutrition programme, translating to longer recovery times.

To our knowledge, there are no comparative studies on cost-effectiveness of CMAM program conducted in similar settings during the COVID-19 pandemic. The reduction in treatment cost per child during the pandemic could be explained by several factors, including treatment protocol modifications and program changes in service delivery. There was a shift from operating nutrition sites from higher- to lower-level health facilities. NGOs shifted to provide nutrition services from hospitals with higher operating costs to standalone nutrition sites and community-level activities with low operating costs (Fig. 2). This reduced the cost associated with running CMAM activities. Additionally, the reduction in visit frequency from weekly to biweekly for SAM children and biweekly to monthly for MAM children likely resulted in reduction in non-RUF consumable supplies and programme operation costs including running generator, transportation of staff to and from nutrition sites. The number of CMAM treatment sites nationally increased during COVID-19, however, compared with before COVID, total CMAM admissions declined at a national level, and those states with the largest declines in admissions also saw reductions in the number of treatment facilities^(15)^. From this analysis, we cannot conclusively deduce that the reduction in unit cost is entirely related to the protocol modification. We observed a wider programme adaptation during COVID including more availability of RUF, increase in caseloads and a shift to lower health facilities. All of these could have affected the observe cost reduction.

The national decline in CMAM admissions is noteworthy because it is opposite to what was observed in the twenty-two counties included in this costing study where admissions increased by 19·8 %. This could be attributed to the low sample size we used in the costing exercise, introducing a potential selection bias. Among the states with the highest levels of acute food insecurity that were included in the costing study, the greatest increases in CMAM enrollment ranged from 1·0 % to 61·7 %. Among the eight states included in the costing study, those states with greater increases in caseload also tended to have larger reductions in the cost per child treated (see online supplementary material, Supplemental Fig. 2). Economies of scale appear to be a main driver of cost in South Sudan, where high enrollment numbers translate to lower per unit costs where fixed costs are distributed over a larger number of children enrolled, thereby reducing cost per child (Fig. 3). Providing patient care irrespective of the number of caseloads requires fixed cost expenditures on health workers, infrastructure, equipment and supervision while variable supplies like medicines and RUF depend on enrollment. Similar studies in Niger and Ethiopia have reported observing the effect of economies of scale in nutrition programmes, where decreases in treatment costs coincide with an increase in programme caseload^(22,30)^.

All studies have limitations, and this study is no exception. First, the reduction in cost per child treated observed may not completely be explained by the COVID-19 protocol modifications adapted by the nutrition cluster, as there were several other factors including increased availability of RUF, a shift to lower-level treatment sites and an increase in caseloads. Additionally, a number of shocks affected programming in South Sudan during this period, including conflict, food insecurity, floods and physical access constraints. It is not possible to attribute changes in enrollments or costs to particular external factors or combinations of factors, and these likely varied by state. Second, we did not account for inflation in our comparison, but expect that costs were relatively stable given that cost reporting was done in United States Dollars, which was more stable compared with the local currency. Third, the results may not be nationally generalised because the sample was a convenience sample that included 28 % of all counties, and often only one of several NGOs working in the county participated. However, it is worth noting that there is no great difference between the NGOs who participated in the costing and those who did not participate in terms of CMAM program implementation and COVID-19 adaptations. Fourth, differences in the quality and completeness of expenditure record keeping, supplies utilisation reporting as well as completeness in completing the questionnaire by the NGOs could have led to the differences in costs observed in states and counties. Fifth, our analysis did not include children who would otherwise have been excluded from admission into the programme due to using MUAC-only criteria. Sixth, our methodology focused on NGO program costs only and did not consider all societal and institutional costs and factors associated with the treatment of a malnourished child such as contributions of the Ministry of Health or costs borne by households. Lastly, given the sensitivity in sharing financial data and the limitation with having quality data in humanitarian settings like South Sudan, we do not completely rule out data omission and incompleteness during sharing from the NGOs.

### Conclusion

This study examined treatment costs for acute malnutrition in South Sudan during COVID-19 in a convenience sample of twenty-two counties. During the COVID-19 period, recovery rates for acute malnutrition improved, and there was no apparent relationship between change in programme cost and recovery rates. The primary costs drivers for acute malnutrition treatment in South Sudan were human resources, ready to use foods and community activities. While the top three costs drivers remained consistent both prior to and during the COVID-19 pandemic, human resource costs declined during COVID-19 while ready to use food costs increased, which is a likely due to the increase in caseloads and increased availability of RUF observed during the COVID period and cannot entirely be attributed to the treatment adaptions in response to COVID-19. Reduced visit frequency, which is efficient for staffing, translated to longer recovery times and consumption of additional ready to use foods. Cost per child enrolled and cost per child recovered were highly correlated with caseload, suggesting that economies of scale are a critical driver for cost-effectiveness in CMAM programs, a finding which has been observed elsewhere. When considering strategies to increase treatment coverage of CMAM treatment programs, this finding suggests that investing in capacity, screening and referral at existing CMAM sites to enable expansion of caseload maybe a preferable strategy to increasing the number of CMAM sites. While such strategies could result in longer transit times and potentially translate to an increased number of defaults, use of community sites and mobile teams could offset these challenges while simultaneously allowing programmes to realise the economies of scale that have been observed to reduce acute malnutrition treatment costs.

## Supporting information

Alier et al. supplementary material Alier et al. supplementary material

## Supplementary material

For supplementary material accompanying this paper visit https://doi.org/10.1017/S1368980023002719
