# Supplementary material for: Impact of COVID-19 program adaptations on costs and cost-effectiveness of community management of acute malnutrition program in South Sudan
Source: Public Health Nutr. 2023 Dec 14;27(1):e15. doi: 10.1017/S1368980023002719 (PMC10830371; doi:10.1017/S1368980023002719)
Supplement: Alier et al. supplementary material [file S1368980023002719sup001.docx]

# SUPPLEMENTAL MATERIALS

**Supplemental Table 1:** Cost categories and ingredients

| Category | Ingredients |
| --- | --- |
| Human Resources * | Health-facility technical health workers |
|  | Health-facility non-technical workers |
|  | State/County IP technical project staff prorated to time |
|  | County IP non-technical project staff prorated to time |
|  | National level technical project staff-prorated to time |
|  | National level non-technical project staff-prorated to time |
|  | Technical staff seconded to MOH |
| Therapeutic food+ | RUTF (per sachet) |
|  | RUSF (per sachet) |
|  | F-75 (per 400g CAN) |
|  | F-100 (per 400g CAN) |
|  | RUTF ration card |
|  | Treatment card (per individual card) |
|  | Stock-control forms (per form) |
| Community activities+ | Trainings/sensitizations of CNVs/BHWs |
|  | Incentives to CNVs/BHWs |
|  | Community mobilization activities (radio talk shows, radio jingles, road shows) |
|  | IPC enhancement-masks, hand sanitizer to CNVs/BHWs |
|  | Transport cost for CNV/BHWs |
|  | Printing of community IEC materials and guidelines |
|  | Printing reporting tools (tally sheets, home visist checklist, referral slips, pens) |
|  | MUAC tape for "Family MUAC" |
|  | Supervision by IP (transport, DSA) of community activities |
|  | Supervision by CHD and SMOH (transport, DSA) of community activities |
|  | Other program management related to community activities |
| Fixed Supplies* | New shelter/space created within the reporting period, including for triage |
|  | Other fixed supplies eg chairs, tables, MUAC tapes, weight boards) |
| National program management+ | Office rent |
|  | Office utilities (security, electricity, water) |
|  | Communication and transport cost |
|  | Adminitrative supplies (paper, pens, printers) |
| Logistics and transportation+ | Transport of personnel between National level and State/county level |
|  | Transport of personnel between State/county level and care sites |
|  | Transport of supplies from supplier to country |
|  | Transport of supplies from National level to State level |
|  | Transport of supplies from State level to county level |
|  | Transport of supplies from County level to treatment sites |
|  | Transport of supplies from SAM or MAM sites to community outreach sites |
|  | Storage of supplies at National level |
|  | Storage of supplies at State level |
|  | Storage of supplies at county level |
| Sub-national program management+ | Office rent |
|  | Office utilities (security, electricity, water) |
|  | Communication and transport cost |
|  | Adminitrative supplies (paper, pens, printers) |
| Medicines and supplies | Vitamin A (per 100,000 IU capsules) |
|  | Amoxicillin (250 mg caps) |
|  | Amoxici.pdr/oral syrup 125mg |
|  | Malaria test (per test) |
|  | Anti-malarial treatment (per full course)-child |
|  | Anti-malarial treatment (per full course)-adult |
|  | LLIN (per piece) |
|  | Albendazole (per 100 mg tablet) |
|  | Mebendazole (per 100 mg tablet) |
|  | Iron/Folate (per 60 mg iron/400ug folic acid) |
|  | I.V kits (cannula+giving set) |
|  | Nasogastric tubes |
|  | syringes |
|  | Sugar water (10% dextrose) |
|  | ReSoMal,42g sachet |
| Infection prevention and control | IPC supplies (jik, mask, hand sanitizer) |
|  | IPC infrastructure (water storage, handwashing facilities) |
| Training and supervision+  (Training items include hall rental, trainer fees, refreshments, stationery, per diems, travel, accommodations) | TOT Training (full training) |
|  | Roll-out training (full training) |
|  | Refresher training |
|  | Sensitization/orientation meetings |
|  | Supervision (by partner staff) |
|  | Supervision (by CHD and SMOH staff) |
| Data and communication+ | Data collection and reporting tools (registers, tally sheets and reporting forms) |
|  | Communication and data sharing (internet, phone, thuray) |
| Evidence generation and assessments+ | Research activities |
|  | Program evaluations |
|  | Assessments (Rapid Needs Assessments, SMART surveys) |

* Fixed cost

+Variable cost

**Supplemental Table 2:** The cost contribution of program components per child recovered by State and Organization Type (in USD)

|  | **Total Costs** | **Human Resources** | **Therapeutic food** | **Community activities** | **Fixed supplies** | **Natl program management** | **Logistics & transport** | **Sub-national program mgmt** | **Medicine & medical supplies** | **IPC** | **Training & supervision** | **Data mgmt & communication** | **Assessments & evidence** |
| --- | --- | --- | --- | --- | --- | --- | --- | --- | --- | --- | --- | --- | --- |
| **By State** | | | | | | | | | | | | | |
| **Central Equatoria (1 county)** | | | | | | | | | | | | | |
| Pre-COVID | 227 | 113.8 | 0.3 | 16.6 | 66.4 | 0.9 | 3.0 | - | 0.2 | 22.6 | 3.0 | 0.1 | - |
| During COVID | 166 | 105.7 | 29.9 | 19.9 | - | - | 6.3 | - | 0.3 | 1.2 | 2.6 | 0.1 | 0.1 |
| Change (%) | -27% | -7% | 9890%+ | 20% | -100% | -100% | 111% | * | 24% | -95% | -14% | 31% | * |
| **Eastern Equatoria (2 counties)** | | | | | | | | | | | | | |
| Pre-COVID | 183 | 25.7 | 57.7 | 5.9 | 0.6 | 63.9 | 7.1 | 9.3 | 6.3 | 0.3 | 3.2 | 1.1 | 1.9 |
| During COVID | 106 | 22.6 | 14.0 | 3.3 | 0.5 | 47.3 | 5.2 | 6.6 | 0.3 | 0.4 | 3.0 | 1.0 | 1.8 |
| Change (%) | -42% | -12% | -76% | -45% | -22% | -26% | -26% | -29% | -95% | 25% | -6% | -10% | -5% |
| **Western Equatoria (4 counties)** | | | | | | | | | | | | | |
| Pre-COVID | 489 | 183.09 | 49.58 | 102.48 | 5.19 | 24.18 | 21.89 | 62.64 | 1.11 | 34.71 | 4.11 | 0.01 | - |
| During COVID | 249 | 107.87 | 44.08 | 57.74 | 2.79 | 4.27 | 6.63 | 3.40 | 0.58 | 19.96 | 1.68 | 0.01 | - |
| Change (%) | -49% | -41% | -11% | -44% | -46% | -82% | -70% | -95% | -48% | -43% | -59% | -30% | * |
| **Jonglei (5 counties)** | | | | | | | | | | | | | |
| Pre-COVID | 168 | 53.7 | 46.0 | 14.9 | 4.3 | 5.0 | 15.6 | 7.7 | 9.8 | 0.3 | 5.7 | 4.5 | 0.5 |
| During COVID | 152 | 43.4 | 68.5 | 10.4 | 4.9 | 3.8 | 7.8 | 3.4 | 5.3 | 0.4 | 2.7 | 1.4 | 0.1 |
| Change (%) | -10% | -19% | 49% | -31% | 15% | -24% | -50% | -56% | -46% | 27% | -52% | -68% | -86% |
| **N. Bahr el Gazal (2 counties)** | | | | | | | | | | | | | |
| Pre-COVID | 79 | 44.0 | 24.6 | 3.2 | 1.3 | 1.4 | 1.58 | 0.5 | 0.4 | 0.2 | 1.3 | 0.6 | - |
| During COVID | 73 | 27.2 | 34.3 | 4.1 | 1.1 | 1.4 | 1.87 | 0.4 | 0.2 | 0.8 | 1.0 | 0.6 | - |
| Change (%) | -6% | -38% | 39% | 27% | -14% | 2% | 18% | -15% | -40% | 304% | -21% | 0% | * |
| **Unity (1 county)** | | | | | | | | | | | | | |
| Pre-COVID | 35 | 22.6 | 2.5 | 1.7 | 1.1 | 0.5 | 2.2 | 2.7 | 0.3 | 0.3 | 0.7 | 0.3 | - |
| During COVID | 39 | 21.2 | 5.6 | 1.8 | 3.0 | 1.8 | 1.2 | 1.9 | 0.4 | 0.8 | 0.5 | 0.5 | 0.5 |
| Change (%) | 10% | -6% | 119% | 2% | 181% | 252% | -46% | -28% | 8% | 137% | -19% | 38% | * |
| **Upper Nile (4 counties)** | | | | | | | | | | | | | |
| Pre-COVID | 341 | 186.2 | 26.0 | 24.0 | 8.70 | 25.1 | 21.2 | 8.8 | 27.3 | 1.3 | 6.8 | 4.8 | 0.8 |
| During COVID | 181 | 105.8 | 8.0 | 17.1 | 2.4 | 14.9 | 13.8 | 4.7 | 6.1 | 1.8 | 3.2 | 2.9 | 0.4 |
| Change (%) | -47% | -43% | -69% | -29% | -72% | -41% | -35% | -46% | -78% | 38% | -53% | -41% | -42% |
| **Warrap (3 counties)** | | | | | | | | | | | | | |
| Pre-COVID | 64 | 30.2 | 9.8 | 9.0 | 2.0 | 1.4 | 1.7 | 0.6 | 6.1 | 0.5 | 0.8 | 0.6 | 1.3 |
| During COVID | 56 | 22.9 | 10.4 | 9.6 | 0.4 | 1.6 | 2.5 | 0.7 | 5.2 | 0.4 | 0.5 | 0.9 | 0.9 |
| Change (%) | -12% | -24% | 6% | 7% | -80% | 21% | 50% | 9% | -14% | -26% | -39% | 61% | -35% |
| **By Organization Type** | | | | | | | | | | | | | |
| **iNGOs (16 counties)** | | | | | | | | | | | | | |
| Pre-COVID | 123 | 52.0 | 23.1 | 10.8 | 8.3 | 6.7 | 4.6 | 5.7 | 4.8 | 3.9 | 1.7 | 0.9 | 0.6 |
| During COVID | 102 | 38.9 | 32.6 | 9.3 | 2.4 | 5.6 | 4.1 | 2.1 | 3.2 | 1.4 | 1.1 | 0.9 | 0.5 |
| Change (%) | -17% | -25% | 41% | -14% | -71% | -17% | -10% | -64% | -32% | -63% | -35% | -5% | -22% |
| **nNGOs (6 counties)** | | | | | | | | | | | | | |
| Pre-COVID | 215 | 70.9 | 39.2 | 27.3 | 7.3 | 11.7 | 27.6 | 5.2 | 6.3 | 0.4 | 11.5 | 7.5 | - |
| During COVID | 147 | 47.8 | 45.7 | 17.9 | 1.6 | 9.7 | 10.1 | 3.6 | 2.1 | 1.1 | 5.9 | 1.5 | - |
| Change (%) | -32% | -33% | 17% | -34% | -78% | -17% | -63% | -31% | -66% | 152% | -49% | -81% | * |

* No reported expenditures or its very minimal.

+ There were massively low quantities of RUF reported by the NGO during the pre-COVID period, that portrays a very low cost per child recovered and hence the very high percent change recorded.

**Supplemental Table 3:** Recovery rate of NGOs compared to overall county-level recovery rate.

|  | **NGO level recovery rate** | | | **overall county level recovery rate** | | |
| --- | --- | --- | --- | --- | --- | --- |
| **County** | **Pre-COVID** | **COVID-19** | **% change** | **Pre-COVID** | **COVID** | **% change** |
| Akobo | 95.9% | 96.4% | 0.5% | 94.8% | 93.2% | -1.6% |
| Aweil South | 85.4% | 90.9% | 5.4% | 85.5% | 88.0% | 2.5% |
| Aweil West | 94.4% | 97.3% | 2.9% | 91.5% | 95.0% | 3.5% |
| Baliet | 70.0% | 94.3% | 24.3% | 86.6% | 95.3% | 8.7% |
| Ezo | 94.8% | 93.4% | -1.4% | 95.1% | 86.9% | -8.2% |
| Fashoda | 96.4% | 97.1% | 0.7% | 95.6% | 95.8% | 0.2% |
| Gogrial East | 86.8% | 86.3% | -0.5% | 91.7% | 94.9% | 3.2% |
| Ikotos | 85.2% | 93.4% | 8.2% | 84.1% | 92.9% | 8.8% |
| Juba | 86.5% | 84.9% | -1.6% | 88.2% | 88.6% | 0.4% |
| Melut | 97.6% | 98.0% | 0.4% | 90.9% | 98.3% | 7.4% |
| Nagero | 95.6% | 99.8% | 4.3% | 98.4% | 100.0% | 1.6% |
| Panyikang | 87.9% | 97.0% | 9.1% | 89.5% | 95.0% | 5.5% |
| Pibor | 69.2% | 93.3% | 24.0% | 94.2% | 94.0% | -0.2% |
| Pochalla | 94.5% | 97.1% | 2.6% | 97.3% | 94.0% | -3.3% |
| Rubkona | 98.0% | 97.7% | -0.3% | 97.3% | 94.4% | -2.9% |
| Tambura | 96.1% | 99.1% | 3.0% | 98.1% | 99.1% | 1.0% |
| Tonj East | 94.2% | 96.9% | 2.7% | 94.2% | 97.2% | 3.0% |
| Tonj North | 91.4% | 91.6% | 0.2% | 94.3% | 92.0% | -2.3% |
| Torit | 90.3% | 95.5% | 5.2% | 91.4% | 95.5% | 4.1% |
| Twic East | 89.3% | 92.4% | 3.1% | 88.2% | 87.3% | -0.9% |
| Uror | 86.5% | 94.1% | 7.6% | 87.7% | 93.2% | 5.5% |
| Yambio | 98.4% | 98.7% | 0.3% | 95.9% | 96.7% | 0.8% |
| Overall average | 90.2% | 94.8% | 4.6% | 92.3% | 94.0% | 1.7% |

**Supplemental Table 4:** Cost Drivers by Program Period and maximum and minimum contribution

|  | **Costs in the Pre-COVID Period** | | | | | | **Cost in the COVID Period** | | | | | |
| --- | --- | --- | --- | --- | --- | --- | --- | --- | --- | --- | --- | --- |
|  | Overall Contribution | | Maximum Contribution | | Minimum Contribution | | Overall Contribution | | Maximum Contribution | | Minimum Contribution | |
|  | Amount ($) | **%** | State* | % | State* | % | Amount ($) | **%** | State* | % | State* | % |
| Total program cost | **17,517,838** |  |  |  |  |  | **22,479,864** |  |  |  |  |  |
| Human resources | **7,125,971** | **40.7%** | Unity | 65% | EES | 14% | **8,361,864** | **37.2%** | CES | 64% | EES | 21% |
| Ready to use foods | **3,271,448** | **18.7%** | EES | 32% | CES | 0.1% | **7,153,807** | **31.8%** | NBeG | 47% | Upper Nile | 4% |
| Community activities | **1,652,419** | **9.4%** | WES | 21% | EES | 3% | **2,154,744** | **9.6%** | WES | 23% | EES | 3% |
| Fixed supplies | **1,086,488** | **6.2%** | CES | 29% | EES | 0.4% | **488,629** | **2.2%** | Unity | 8% | CES | 0% |
| National program management | **952,262** | **5.4%** | EES | 35% | CES | 0.4% | **1,265,873** | **5.6%** | EES | 45% | CES | 0% |
| Logistics | **923,748** | **5.3%** | Jonglei | 9% | CES | 1% | **1,012,883** | **4.5%** | Upper Nile | 7% | NBeG | 3% |
| Training and supervision | **743,523** | **4.2%** | WES | 13% | CES | 0% | **469,837** | **2.1%** | EES | 6% | CES | 0% |
| Sub-national program management | **648,727** | **3.7%** | Warrap | 9% | CES | 0.1% | **644,708** | **2.9%** | Warrap | 9% | CES | 0.2% |
| Medicine and supplies | **467,541** | **2.7%** | CES | 10% | EES | 0.2% | **290,314** | **1.3%** | WES | 8% | Jonglei | 0.2% |
| Infection prevention & control | **361,445** | **2.1%** | Jonglei | 3% | WES | 1% | **351,750** | **1.6%** | EES | 3% | WES | 1% |
| Data and communication | **215,377** | **1.2%** | Jonglei | 3% | WES | 0% | **201,376** | **0.9%** | Warrap | 2% | WES | 0% |
| Evidence generation | **68,890** | **0.4%** | Warrap | 2% | WES, CES & NBeG | 0% | **84,080** | **0.4%** | EES | 2% | WES & NBeG | 0% |

**Supplemental Figure 1**: Change in caseload and cost per child enrolled in CMAM programs during COVID-19 in South Sudan (compared to pre-COVID)
